# Spectrum of Copy Number Variants in Fetal Congenital Heart Disease and Their Clinical Implications: A Retrospective Study from a Tertiary Care Center

**DOI:** 10.3390/diagnostics16060854

**Published:** 2026-03-13

**Authors:** Meiying Cai, Na Lin, Meimei Fu, Yanting Que, Miao Zheng, Liangpu Xu, Hailong Huang

**Affiliations:** 1 Medical Genetic Diagnosis and Therapy Center, Fujian Maternity and Child Health Hospital College of Clinical Medicine for Obstetrics & Gynecology and Pediatrics, Fujian Medical University, Fuzhou 350001, China; caimeiying@fjmu.edu.cn (M.C.); linna1088@fjmu.edu.cn (N.L.); fumeimei2009@126.com (M.F.); queyanting1998@163.com (Y.Q.); xiliangpu@fjmu.edu.cn (L.X.); 2 Fujian Key Laboratory for Prenatal Diagnosis and Birth Defect, Fuzhou 350001, China; 3 National Key Obstetric Clinical Specialty Construction Institution of China, Fuzhou 350001, China; 4 Department of Clinical Laboratory, Fujian Maternity and Child Health Hospital College of Clinical Medicine for Obstetrics & Gynecology and Pediatrics, Fujian Medical University, Fuzhou 350001, China

**Keywords:** fetal congenital heart disease, chromosomal microarray analysis, fetal echocardiography, pathogenic copy number variation

## Abstract

**Background/Objectives:** This study assesses the genetic basis of fetal congenital heart disease (CHD), which exhibits a complex etiology, by using chromosomal microarray analysis (CMA); it also elucidates perinatal outcomes and postnatal development to support prenatal diagnosis and genetic counseling. **Methods:** Pregnant women (n = 1195) who were diagnosed with fetal CHD based on echocardiography were selected along with those having an interventional prenatal diagnosis, all of whom underwent CMA. Depending on the gestational age, amniotic fluid or umbilical cord blood samples were collected. Patients were included if they were diagnosed with fetal CHD based on echocardiography. Those who could not consent to amniocentesis or umbilical vein puncture or who had contraindications for amniocentesis or umbilical vein puncture were excluded. Patients were studied until May 2025. **Results:** Of the 1195 fetuses with CHD, 140 had pathogenic copy number variation (pCNV). The pCNV detection rate in cases with a single CHD was 3.17%, whereas it was 13.51% in the group with multiple CHDs. The detection rate for pCNVs in patients with extracardiac abnormalities was 28.62%. The fetal and postnatal mortality rates were highest for fetuses with multiple CHDs. The survival rate was highest for fetuses with a single CHD. Early detection of CHD and timely genetic testing can inform clinical management of CHD-affected pregnancies; however, larger prospective studies are needed to establish their impact on perinatal outcomes. **Conclusions**: CMA provides valuable information for genetic counselling, as it identifies pathogenic variants associated with CHD. However, prognostic predictions should consider multiple clinical factors.

## 1. Introduction

Congenital heart disease (CHD) is one of the most common birth defects [1,2,3]. Prenatal echocardiography can be used to detect CHD and determine the type of CHD in more than 85% of fetuses [4]. Because of advancements in surgical techniques, most children with CHD achieve a good prognosis through surgery. However, some fetuses with complex pathogenic copy number variations (pCNVs) may develop problems, such as developmental delay, special facial features, intellectual disability, and low immunity after birth, making it impossible for these children to receive effective treatment and posing a huge burden on affected families and the broader society [5]. Therefore, fetal screening for the diagnosis of CHD should not be limited to ultrasound imaging. Genetic testing should also be conducted to better assess the prognosis of patients with CHD and reduce the birth defect rate of fetuses.

The pathogenesis of CHD involves multiple interacting factors. Genetic factors are crucial in this process; however, the specific pathogenic mechanisms have not been clearly defined [6]. Genetic causes of CHD include chromosomal abnormalities, such as trisomy 13, trisomy 18, trisomy 21, and Turner syndrome, as well as structural abnormalities including 22q11 microdeletion [7]. pCNVs significantly affect the neurological development, quality of life, and life expectancy of children with CHD [8]. Therefore, pCNV detection during prenatal assessment is critical. Chromosome microarray analysis (CMA) can detect a wide range of abnormalities, from aneuploidy to small microdeletions and/or microduplications. The American College of Obstetricians and Gynecologists and the Society for Maternal-Fetal Medicine have also recommended using CMA for the prenatal diagnosis of one or more fetal structural abnormalities [9]. CMA has a higher diagnostic yield for CNVs in fetal CHD. Moreover, there are practical constraints of whole-exome sequencing (WES; e.g., cost, turnaround time) in prenatal contexts.

In this study, we aimed to retrospectively analyzed fetuses with CHD who underwent both echocardiography and CMA at our hospital to assess the genetic causes of CHD and provide a scientific basis for fetal prognosis assessment, genetic counseling of pregnant women, and fertility guidance. pCNVs were detected at a higher rate in cases with a single CHD than those with multiple CHDs. This was accompanied by higher fetal and postnatal mortality rates for fetuses with multiple CHDs.

## 2. Materials and Methods

### 2.1. Patient Data

We selected 1195 fetuses from Fujian Provincial Maternal and Child Health Hospital who underwent fetal echocardiography for CHD and received an interventional prenatal diagnosis from January 2020 to December 2024. CMA was performed for all fetuses. Pregnant women were included if fetal CHD was confirmed via prenatal ultrasound screening performed by at least two experienced maternal-fetal specialists, using standardized diagnostic criteria (e.g., International Society of Ultrasound in Obstetrics and Gynecology [ISUOG] guidelines). Cases were excluded if they (1) lacked consent for amniocentesis/umbilical vein puncture, (2) had contraindications to these procedures, or (3) showed discordant ultrasound diagnoses between observers. All included cases required a unanimous CHD diagnosis by the specialist team. The gestational age was 21 ± 3 weeks (16–34 weeks), and the maternal age was 29 ± 6 years (19–45 years). Amniocentesis was performed on pregnant women with gestational ages ranging from 16 to 24 weeks. Umbilical vein puncture was performed through the abdomen to draw umbilical blood for those with a gestational age ≥ 24 weeks. All participants signed an informed consent form for interventional prenatal diagnosis. This study was approved by the Ethics Committee of Fujian Maternal and Child Health Hospital (protocol code 2014042).

### 2.2. Diagnostic Criteria for Fetal CHD

Prenatal ultrasound diagnosis of CHD was made according to the screening guidelines for fetal echocardiography issued by the ISUOG [10]. Fetal echocardiography was performed in accordance with current international guidelines [11]. Voluson E8 (GE Healthcare, Mascot, Austria) and Voluson E10 (GE Healthcare, Austria) color Doppler ultrasound diagnostic devices, with a frequency range of 3–5 MHz, were used as convex array probes. Grade III ultrasound is a comprehensive fetal echocardiography procedure performed at specialized prenatal diagnostic centers. It involves systematic cardiac evaluation (including four-chamber view, outflow tracts, aortic/ductal arches, and venous connections), spectral/color Doppler studies for hemodynamic assessment, and concurrent screening for extra-cardiac anomalies. Depending on the presence of single or multiple CHDs and whether they were accompanied by extracardiac abnormalities, patients were divided into three groups: single CHD, multiple CHD, and CHD combined with extracardiac abnormalities. Single CHD refers to single congenital heart defects involving a single primary cardiac anomaly, such as simple transposition of the great arteries without additional cardiac or extracardiac defects. The multiple CHD group included patients having congenital heart defects with two or more primary cardiac anomalies such as tetralogy of Fallot (TOF), which comprises a ventricular septal defect (VSD) that overrides the aorta, right ventricular outflow tract obstruction, and right ventricular hypertrophy. CHD with extracardiac abnormalities represents the congenital heart defects that were accompanied by one or more major structural or syndromic anomalies outside the cardiovascular system, exemplified by a VSD with cleft palate.

### 2.3. CMA

AF samples were processed following standard operating procedures using a CytoScan 750k Chip Reagent Kit (Affymetrix, Santa Clara, CA, USA). Data were analyzed using ChAS interpretation software version 4.2. Based on data from public databases—including the DECIPHER database related to disease-causing CNVs, the OMIM database of known genetic diseases and disease-causing genes in humans, the DGV database of polymorphic variations, the UCSC (Genome Browser and PubMed) database for analyzing gene content and function in CNVs, and internal reference databases (including the CAGdb database of the Affymetrix chip platform and the ISCA database of the International Consortium for Cell Genomic Chips)—the results of the ChAS software analysis were analyzed to determine the pathogenicity of the CNVs. CNVs were classified according to the latest guidelines of the American College of Medical Genetics and Genomics (ACMG, 2019) as pathogenic, likely pathogenic, of unknown clinical significance, benign, or likely benign. All variants of unknown significance (VUS) classifications were based on the 2019 ACMG/AMP guidelines and subsequent updates. Following our internal review process, variants with conflicting evidence were reviewed by at least two clinical geneticists to reach a consensus, and unresolved cases were referred to external databases.

### 2.4. Pregnancy Outcomes

All enrolled pregnant women underwent systematic follow-up via outpatient visits or structured telephone interviews at predefined intervals (e.g., 1, 3, and 6 months post-delivery). Pregnancy outcomes, neonatal birth defects, and infant prognoses were prospectively recorded. To address potential loss-to-follow-up bias, baseline characteristics were compared between cases with complete follow-up and those lost to follow-up (no significant differences were observed in maternal age, gestational age at diagnosis, or CHD severity, *p* > 0.05). Given the limited missing data for primary outcomes (<5% of cases), formal imputation was not performed for the final analysis.

### 2.5. Statistical Analysis

Statistical analysis was conducted using SPSS software (version 21.0; IBM, Armonk, NY, USA). Count data were expressed as frequencies and percentages, and comparisons between groups were performed using the chi square test. Statistical significance was set at *p* < 0.05. The sample size for this study was based on practical considerations, as this was an exploratory study.

## 3. Results

### 3.1. Type of Fetal Heart Structure Malformation

Among the 1195 fetuses, 662 (55.40%) had a single CHD, 222 (18.58%) had multiple CHDs, and 311 (26.03%) had CHD with extracardiac abnormalities (Table 1). In the single CHD group, the most common anomaly was VSD in 444 cases (37.15%), followed by aortic arch anomaly in 140 cases (11.72%) and persistent left superior vena cava in 51 cases (4.27%). Among patients with CHD accompanied by extracardiac abnormalities, multiple system abnormalities were the most common (n = 74, 6.19%), followed by skeletal system abnormalities (n = 42, 3.51%), the absence of nasal bone (n = 27, 2.26%), and nuchal translucency thickening (n = 27, 2.26%).

### 3.2. Results of CMA for CHD Fetuses

**Figure 1:**
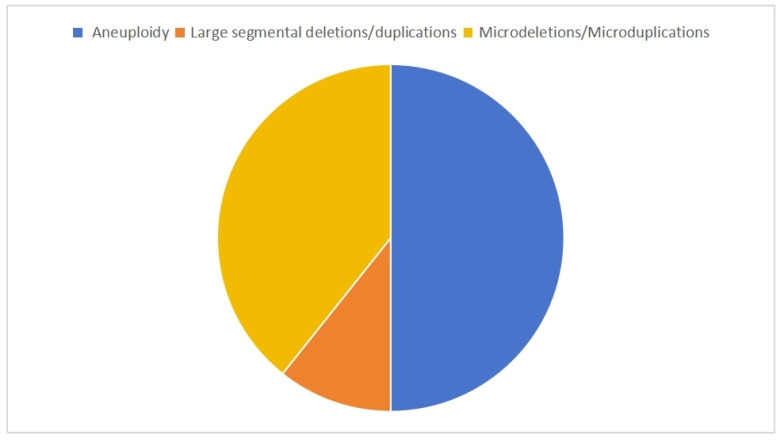
Classification of pathogenic copy number variations.

Pathogenic CNVs were detected in 11.7% (140/1195) of fetuses with CHD (Table 2), comprising three main categories: (1) 70 aneuploidies (trisomy 21 [n = 25], trisomy 18 [n = 24], and 45X [n = 10] were most frequent); (2) 15 large segmental CNVs; and (3) 55 microdeletions/duplications, predominantly at 22q11.2 (n = 17) and 15q11.2 (n = 6) (Figure 1). CMA identified both well-established CHD-associated CNVs (e.g., 22q11.2 involving *TBX1*/*CRKL*, 7q11.23 with ELN) and potential candidates (e.g., 15q11.2, 16p13.11). Additionally, 2.1% (25/1195) of cases showed VUSs, spanning 0.15–58.06 Mb across multiple chromosomes, with 72% (18/25) occurring in cases with single CHD.

### 3.3. Detection of pCNVs in Different Types of CHD Fetuses

**Figure 2:**
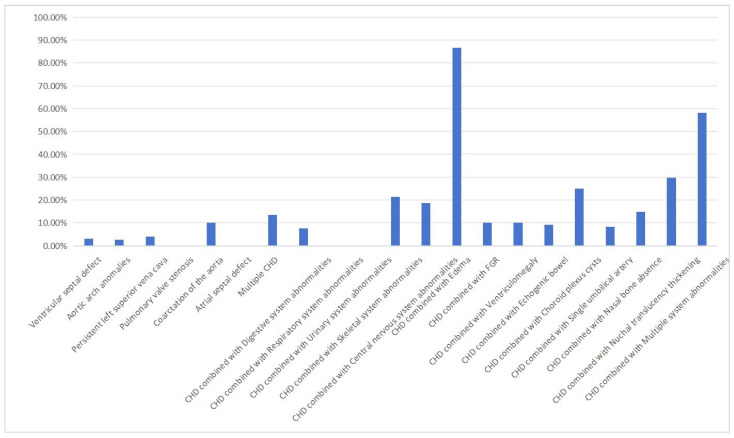
Detection of pathogenic copy number variations in fetuses with different types of congenital heart disease (CHD).

The detection rate of pCNVs was 3.17% (21/662) in patients with a single CHD, 13.51% (30/222) in the group with multiple CHDs, and 28.62% (89/311) in those with CHD and extracardiac abnormalities. The detection rates of pCNVs varied significantly among the three groups (χ^2^ = 133.01, *p* < 0.0001). Among patients with a single CHD, the detection rate of pCNVs was highest for those with aorta coarctation (10.00%, 1/10), followed by those with aortic arch anomalies (4.29%, 6/140) and persistent left superior vena cava (3.90%, 2/51). Among patients with CHD accompanied by extracardiac abnormalities, the detection rate of pCNVs was highest for those with edema (86.67%, 13/15), followed by those with multiple system abnormalities (58.11%, 43/74) and nuchal translucency thickening (29.63%, 8/27) (Figure 2).

### 3.4. Pregnancy Outcomes

Among the 1195 cases with CHD, 90 were lost to follow-up, giving a follow-up success rate of 92.47% (1105/1195). Among those that completed follow-up, 186 cases underwent pregnancy termination (among which 133 cases carried pCNVs), 51 represented stillbirths, 5 denoted perinatal deaths, and the remaining 863 cases were live births. Statistical analysis revealed significant outcome differences by CHD subtype (all *p* < 0.001). Fetuses with CHD plus extracardiac abnormalities had a significantly higher termination rate (40.00%, 116/290) than those with single CHD (8.59%, 70/815, *p* < 0.001). Fetuses with multiple CHDs showed elevated stillbirth/perinatal mortality (12.62%, 26/206) compared to those with an single CHD (0.49%, 3/609, *p* < 0.001). Pathogenic CNVs were associated with higher rates of pregnancy termination, while CNVs of VUS were more prevalent in live births (Table 3).

## 4. Discussion

CMA can detect genetic variations related to CHD in fetuses. CMA has the advantages of superior sensitivity and faster turnaround time compared to traditional chromosome karyotype analysis. pCNVs were detected in 140 of the 1195 cases with CHD included in this study. Of those with pCNVs, 70 had aneuploidies, 15 had large segmental deletions/duplications, and 55 had microdeletions/microduplications. The detection rate of pCNV was higher in patients with CHD and extracardiac abnormalities than in patients with only CHD [12]. However, Luo et al. [13] found no significant difference in the pCNV detection rate between patients with simple and multiple CHDs. The results of this study showed that pCNV detection rates were higher in the group with both CHD and extracardiac abnormalities and the group with multiple CHDs when compared with the single CHD group. These differences in pCNV detection rates among the three groups were statistically significant. Multiple CHD cases and CHD combined with extracardiac abnormalities were more likely to be accompanied by pCNVs, meriting attention in clinical practice and indicating that CMA testing should be promptly conducted.

Among the key genes affected by 22q11.2 deletion/duplication, *TBX1* is a core regulatory factor involved in the development of the cardiac outflow tract and the aortic arch. It affects the separation of major arteries by regulating the proliferation of pharyngeal arch mesenchymal cells [14]. Among patients with 22q11.2 deletion, more than 70% had cardiac malformations (such as TOF and persistent truncus arteriosus). *Tbx1* deletion reproduces these phenotypes in a mouse model. As a linker protein, CRKL mediates neural crest cell migration via the FGF8 signaling pathway. Its absence leads to abnormal development of the cardiac outflow tract. The mouse Crkl-knockout model shows conotruncal artery malformation, overlapping with the human 22q11.2 deletion phenotype [15]. *ELN* (elastin gene) deletion in the 7q11.21 and 11q11.23 regions reduces the number of elastic fibers in the vascular wall, resulting in stenosis of large blood vessels, aortic valve stenosis, and pulmonary artery stenosis [16]. Among the key genes affected by 1p36 deletion, *SKI* is a negative regulator of the TGF-β signaling pathway. Its absence leads to excessive activation of TGF-β signaling, which affects atrioventricular cushion remodeling and interventricular septal closure [17]. 11q24.2q25 deletion leads to the regulation of endothelial-mesenchymal transition by ETS1, which is crucial for the formation of heart valves and ventricular septa. An insufficient single-copy dose of *ETS1* is associated with VSD and TOF [18]. Possible microdeletions/microduplications associated with CHD include: 15q11.2 microdeletion (*CYFIP1*), 16p13.11 microdeletion/microduplication (*MYH11*), 16p11.2 microdeletion/microduplication (*TBX6*), and 1q21.1 microduplication (*GJA5*). The detection rate of 15q11.2 microdeletion was relatively high in patients with CHD. However, the underlying pathogenic mechanisms remain unclear. CYFIP1 may affect cardiac development through the Rac1 signaling pathway [19]. The *MYH11* gene in the 16p13.11 deletion/duplication region is mainly associated with aortic diseases, but this variant has been detected in some patients with CHD (such as those with aortic stenosis) [20]. The *TBX6* gene, involved in segmental development, is present in the 16p11.2 deletion/duplication region. Its absence may result in abnormalities of the spine and heart [21]. Microduplication of the *GJA5* gene (encoding connexin 40) in 1q21.1 is clearly associated with atrial arrhythmias and septal defects [22]. However, the associations of other pCNV variations (such as the 6q26q27 and 4q22.2q24 deletions) with CHD merit further verification.

Previous studies have shown that 3–14% of single CHD cases may involve pCNVs [23,24]. Among single CHDs, VSDs are the most predominant type, followed by aortic arch anomalies. Because of their high prevalence, the genetic abnormalities and clinical outcomes associated with these lesions may overshadow other less common CHDs in this category. In this study, single VSD accounted for 37.15% (n = 444) of all CHD cases, with a pCNV detection rate of 3.17%; this result is consistent with previous reports [25]. However, the predominance of VSDs and aortic arch anomalies may mask the genetic and phenotypic diversity of rarer CHD subtypes, warranting further investigation into their distinct molecular mechanisms and clinical implications. In this study, the detection rate of pCNVs in the single CHD group was highest for those with aortic coarctation (10.00%, 1/10). Coarctation of the aorta refers to a congenital heart disease characterized by local narrowing of the aortic arch, accounting for 5–7% of all fetuses with CHD [26]. Previous genetic etiological studies have shown that the detection rate for fetal coarctation of the aorta when using CMA varies from 0% to 100% [27,28,29]. A possible reason for these inconsistencies may be heterogeneity in the study population, as the distribution of genetic causes of CHD varies among different races and regions. Although the detection rate of pCNVs in single CHD is low, it significantly increases when accompanied by extracardiac abnormalities. In a study of newborns with CHD, extracardiac abnormalities, and neurodevelopmental delays, the detection rate of CMA was 17–53% [30,31]. Our detection rate of pCNVs in CHD combined with extracardiac abnormalities was 28.62% (89/311), similar to the rate reported for newborns with CHD. Similarly, Jansen et al. [32] conducted a meta-analysis and found that the incidence of pCNVs was high in fetuses with multiple CHDs. Reversed A-waves in the ductus venosus and nuchal translucency thickening are closely related to the occurrence of CHD [33]. In fetuses with CHD and edema, the detection rate of pCNVs by CMA can be as high as 40–60% [27]. In fetuses with CHD showing translucency thickening, the detection rate of pCNVs by CMA is 20–30% [27]. In this study, the detection rate of pCNV in patients with CHD combined with edema (86.67%, 13/15) was the highest, followed by those with CHD combined with multiple system abnormalities (58.11%, 43/74) and CHD combined with nuchal translucency thickening (29.63%, 8/27). This result is consistent with those of the previous studies. Therefore, when CHD is detected prenatally and is accompanied by other systemic abnormalities, especially edema or nuchal translucency thickening, patients should visit a genetic counseling clinic.

In clinical settings, the pathogenicity of CNVs is evaluated based on the gene content, size of CNVs, and gene structure [34]. Some CNVs have unclear clinical significance, and it is difficult to accurately classify them. However, through continuous research, their pathogenicity may be discovered in the future [35,36]. Twenty-five cases with VUSs were identified in this study (2.09%, 25/1195). VUSs increase the difficulty of prenatal genetic counseling, causing great anxiety for parents. These variants should be evaluated in combination with the CNV test results of the parents. The influence of incomplete penetrance and clinical phenotypic differences should also be considered. While we identified VUSs in this study, it was not possible to provide further guidance for clinical consultations because the parents of the fetus did not consent to perform the kinship CMA test. Although limited by cohort size, our data indicate a potential association between pathogenic CNVs and higher pregnancy termination rates, whereas CNVs of VUS were more frequent in live births. Long-term follow-up is warranted to assess the clinical implications of VUS.

In this retrospective study, most fetuses with a single CHD had live births with generally good postnatal conditions, especially those with VSD. For fetuses with multiple CHDs and those with both CHD and extracardiac abnormalities, the parents often choose to terminate the pregnancy after multidisciplinary prenatal consultations. The pregnancy termination rate was highest for fetuses with CHD and edema. Fetal and postnatal mortality rates were highest for fetuses with multiple CHDs. In this study, the pregnancies for 133 fetuses carrying pCNVs were terminated. During follow-up, there were five live births with pCNVs (one case of 15q11.2 microdeletion, two cases of 1q21.1 microdeletion/microduplication, and two cases of 2p16.3 microdeletion). 15q11.2 microdeletion is associated with neurodevelopmental disorders, such as autism and schizophrenia; however, the penetrance is low (approximately 10%), and most carriers are asymptomatic [37]. The phenotypic spectrum of 1q21.1 microdeletion/microduplication is extensive, ranging from normal intelligence to congenital malformations or mental disorders [38]. The 2p16.3 microdeletion is associated with autism and schizophrenia; however, its expression is incomplete [39]. The subsequent development of the abovementioned five live-born infants needs to be closely monitored over a long period to identify any abnormalities and revise the recommendations for consultation accordingly. This finding indicates the complexity of prenatal pCNV testing in clinical practice. The coexistence of high termination rates and live births underscores the need for more precise genetic counseling, a more comprehensive follow-up system, and attention to socio-psychological factors. In the future, it will be necessary to achieve a balance between technological capabilities and ethical responsibilities to prevent the unnecessary termination of pregnancies due to “technological determinism”.

This study has several limitations. First, as this was a retrospective study, pedigree verification was not conducted for all VUS cases. Such cases pose a challenge for genetic counseling. After searching databases and the relevant literature, genetic counselors should interpret and explain the test results for VUSs. Based on the full understanding of the pregnant woman and her family, unnecessary terminations of pregnancies should be minimized. Second, the average follow-up period for live-born infants in this study was only 6 months. No follow-up was performed during early childhood. However, the follow-up period could be extended to include early childhood. Third, single-gene mutations play a significant role in CHD [40,41,42]. WES was not conducted for cases with negative CMA results. This may have led to the omission of cases caused by single-gene mutations. With advancements in WES technology and improvements in genetic maps, an increasing number of pathogenic variations have been discovered. This has facilitated the prenatal diagnosis and etiological research of fetuses with CHD, allowing more detailed consultations for pregnant women and the formulation of evidence-based pregnancy and treatment plans [43]. Fourth, a notable limitation of our study is the inability to fully assess the relationship between genetic abnormalities and postnatal outcomes. Future prospective studies with long-term follow-up are needed to clarify how specific genetic abnormalities influence survival and postnatal complications in CHD-affected pregnancies. Fifth, while our statistical approach provided valid primary analyses, the study was underpowered for comprehensive multivariate adjustments (e.g., maternal age, parity) and subgroup analyses. This precluded the examination of potential effect modifiers and confounders that may refine clinical interpretations. Future studies with larger cohorts should incorporate these advanced analytical methods to strengthen causal inference. Sixth, as this study was conducted at a single institution, our findings may be influenced by local patient demographics, clinical practices, and referral patterns. Our cohort primarily comprised individuals of insert specific ethnic/geographic background. Given known variations in genetic architecture across populations, the applicability of our genotype-phenotype correlations to other ethnic groups warrants cautious interpretation and future investigation in diverse cohorts. Finally, the clinical significance of third-trimester ultrasound examinations for detecting late-emerging CHD is uncertain. Specifically, certain conditions, such as rhabdomyomas, pulmonary stenosis, ventricular septal defects, and mild aortic coarctation, may only become apparent during later gestation. Our study’s design may have hindered the detection of late-emerging anomalies because of the timing of follow-up scans [44].

## 5. Conclusions

In summary, the genetic factors contributing to fetal CHD are complex, and comprehensive assessments cannot rely solely on prenatal ultrasound. CMA offers valuable diagnostic utility by detecting pCNVs associated with CHD. Our findings support the consideration of CMA for the genetic evaluation of fetuses with CHD; however, its clinical application should account for the availability of regional healthcare resources. While early detection through CMA may inform pregnancy management and genetic counseling, we emphasize the need for prospective multicenter studies to validate the following factors: (1) the diagnostic yield of CMA across diverse CHD subtypes (single vs. non-single), and (2) its long-term impact on perinatal decision-making and outcomes. Prognostic interpretations should integrate CMA results with detailed phenotyping and family history.

## Figures and Tables

**Table 1:** Type of congenital heart disease in 1195 fetuses.

Main Category	Subcategory	Cases (%)
Single CHD	VSD	444 (37.15%)
	Aortic arch anomalies	140 (11.72%)
	Persistent left superior vena cava	51 (4.27%)
	Pulmonary valve stenosis	16 (1.34%)
	Coarctation of the aorta	10 (0.84%)
	Atrial septal defect	1 (0.08%)
Multiple CHD	-	222 (18.58%)
CHD combined with extracardiac abnormalities	Digestive system abnormalities	13 (1.09%)
	Respiratory system abnormalities	10 (0.84%)
	Urinary system abnormalities	22 (1.84%)
	Skeletal system abnormalities	42 (3.51%)
	Central nervous system abnormalities	16 (1.34%)
	Edema	15 (1.26%)
	FGR	20 (1.67%)
	Ventriculomegaly	10 (0.84%)
	Echogenic bowel	11 (0.92%)
	Choroid plexus cysts	12 (1.00%)
	Single umbilical artery	12 (1.00%)
	Nasal bone absence	27 (2.26%)
	Nuchal translucency thickening	27 (2.26%)
	Multiple system abnormalities	74 (6.19%)

Percentages for subcategories (e.g., VSD 37.15%) are calculated against the total cohort (N = 1195), not within the subgroup.

**Table 2:** Prevalence of pathogenic, likely pathogenic, and VUS CNVs across CHD Subtypes.

Subtype	Presence of All Types of CNVs (Number, %)	Pathogenic CNVs (Number, %)	Likely Pathogenic CNVs (Number, %)	CNVs of Uncertain Significance (Number, %)
Single CHDs	39 (23.64%)	16 (9.70%)	5 (3.03%)	18 (10.91%)
Multiple CHDs	30 (18.18%)	26 (15.76%)	4 (2.42%)	0 (0%)
CHDs with extra-cardiac anomalies	96 (58.18%)	88 (53.33%)	1 (0.61%)	7 (4.24%)

Percentages for subcategories are calculated against the total types of CNVs.

**Table 3:** Association between CNV classification and pregnancy outcomes in fetuses with congenital heart defects.

Outcome	All Types of CNVs (Number, %)	Pathogenic CNVs (Number, %)	Likely Pathogenic CNVs (Number, %)	CNVs of Uncertain Significance (Number, %)
Termination of pregnancy	136 (83.95%)	129 (79.63%)	4 (2.47%)	3 (1.85%)
Still birth	3 (1.85%)	1 (0.62%)	1 (0.62%)	1 (0.62%)
Live birth	23 (14.20%)	0 (0%)	5 (3.09%)	18 (11.11%)
Post-natal death	0 (0%)	0 (0%)	0 (0%)	0 (0%)

Percentages for subcategories are calculated against the total pregnancy outcomes in fetuses with all types of CNVs.

## Data Availability

The datasets analyzed during the current study are not publicly available due to institutional restrictions but are available from the corresponding author upon reasonable request. All aggregated results are included in this published article.

## Abbreviations

The following abbreviations are used in this manuscript:

ACMG	American College of Medical Genetics and Genomics
CHD	Congenital heart disease
CMA	Chromosomal microarray analysis
CNV	Copy number variation
pCNV	Pathogenic copy number variation
VSD	Ventricular septal defect
